# Capability–environment configurations driving exploratory innovation in art entrepreneurs: A mixed-methods configurational study

**DOI:** 10.1371/journal.pone.0348315

**Published:** 2026-05-04

**Authors:** Zhaohua Chai, Na Yang

**Affiliations:** 1 College of Performing and Fine Arts, China Three Gorges University, Yichang, China; 2 School of Art and Design, Baise University, Baise, China; Universidade Europeia, Lisboa, PORTUGAL

## Abstract

Exploratory innovation has become a critical factor for art entrepreneurs to remain competitive in dynamic environments. However, existing studies predominantly focus on high-tech or resource-rich firms, offering limited insight into how creative entrepreneurs with constrained resources achieve such innovation. To address this gap, this study integrates dynamic capabilities theory and open systems theory to examine the complex antecedents of exploratory innovation in China’s cultural and creative industries. Based on survey data from 203 art entrepreneurs across various creative sectors, we employ a mixed-method approach combining necessary condition analysis (NCA) and fuzzy-set qualitative comparative analysis (fsQCA). The findings reveal that no single condition can be regarded as a strictly necessary condition for the outcome. Moreover, three distinct configurations lead to high levels of exploratory innovation: internally driven capability alignment, external opportunity activation, and sensing-response adaptation in the face of technological turbulence. This study contributes to the literature by extending the application of dynamic capabilities and open systems theory to the creative entrepreneurship domain and by demonstrating the methodological value of integrating NCA and fsQCA.

## Introduction

The digital economy and the rise of cultural consumption have expanded the scale and visibility of the cultural and creative industry, making it an increasingly significant economic and social force [1,2]. Within this evolving landscape, arts entrepreneurs play a key role in translating creative ideas into cultural products and shaping emerging cultural identities. Yet the rapid shifts in consumer preferences, intensified competition, and the pressure to operate across diverse digital platforms have also created new forms of precarity for these creators [3,4]. Many face unstable income, limited resources, and a narrowing space for experimentation as they strive to balance artistic expression with commercial demands. In this context, exploratory innovation offers a promising means of addressing these challenges [5,6]. By encouraging experimentation with new aesthetic forms, technologies, and market approaches, exploratory innovation enables arts entrepreneurs to adapt to uncertainty, expand creative possibilities, and build more resilient development paths [2]. Therefore, strengthening exploratory innovation is essential for supporting both the sustainability and the cultural vitality of the creative sector.

To promote innovation in arts enterprises, scholars have already undertaken some initial exploratory research. For example, García-Muiña et al. [7] used thematic analysis to propose a systematic innovation management model, aiming to create the right conditions to spark new ideas and provide incentives for innovation in exhibition halls. Scuotto et al. [8] examined the impact of social media platforms on agile innovation in the context of digital transformation within the Italian fashion industry. Liu [9] explored the role of government policies in fostering innovation within the creative industries from a macro-dynamic perspective. While these studies each contribute valuable insights, they fall short of fully capturing the impact mechanisms arising from the interplay of internal and external factors when explaining complex innovation behaviors. Innovation in the creative industries is a complex system, closely tied to an organization’s internal resource base and capability structure, while also being influenced by external factors such as institutional environments, market dynamics, and technological changes [2,10].

Furthermore, the methodological choices in existing empirical studies also hinder a deeper understanding of exploratory innovation. Much of the research relies on linear regression or structural equation modeling, approaches that emphasize isolated effects between variables [11–13]. Because these techniques assume relatively stable and linear causal structures, they rarely capture how multiple conditions interact in nonlinear or complementary ways to influence innovation outcomes [8,14,15]. As a result, the findings produced are often fragmented, leaving the underlying pathways through which organizations pursue innovation in uncertain environments insufficiently explained. These limitations underscore the need for an integrated analytical framework that considers both internal capabilities and external institutional pressures, enabling a more comprehensive understanding of innovation behavior in dynamic contexts.

To address these theoretical and methodological limitations, this study develops an integrative framework that combines dynamic capabilities theory and open systems theory, providing a more comprehensive basis for understanding how internal adaptive capacities interact with external environmental conditions to influence exploratory innovation [16–18]. Dynamic capabilities theory explains how art entrepreneurs sense opportunities, mobilize technological resources, and adjust strategic orientations, whereas open systems theory emphasizes the influence of technological change, institutional pressures, and market dynamics. Integrating these perspectives allows for a more complete analytical view.

Methodologically, this study employs a mixed-method configurational approach that integrates necessary condition analysis (NCA) with fuzzy-set qualitative comparative analysis (fsQCA). NCA offers quantitative rigor in identifying potential boundary conditions for high-level exploratory innovation [19], while fsQCA reveals multiple, equifinal causal configurations that reflect the nonlinear and asymmetric nature of innovation processes in creative industries [20–22]. By combining a theoretically grounded integrative perspective with complementary analytical techniques, this study offers a coherent response to the fragmentation and linear assumptions that have limited prior research and provides a more nuanced understanding of how capability–environment alignments shape exploratory innovation among art entrepreneurs.

## Theoretical framework and model construction

### Literature review

Exploratory innovation refers to the process through which organizations develop entirely new products, services, or business models by breaking through existing knowledge, technology, or market frameworks in uncertain environments [23,24]. Unlike exploitative innovation, exploratory innovation carries higher levels of uncertainty and risk, but it also offers organizations the opportunity to seize market advantages and technological leadership [23–25]. Its core components include: (1) the acquisition and application of breakthrough knowledge; (2) high risks and uncertainty throughout the innovation process; and (3) the potential for long-term strategic value, especially in the context of evolving markets and technologies [24].

Scholars have explored how to promote exploratory innovation from various theoretical perspectives. Zhang and Luo [26] examined the role of organizational network capital in driving exploratory innovation based on network dynamics, while Slavova and Jong [27] focused on organizational learning theory to reveal the relationship between university alliances and corporate exploratory innovation output. Zhu et al. [28] employed job demands theory to analyze why executive job demands may negatively affect a company’s exploratory innovation. Additionally, Qin et al. [29] integrated dynamic capabilities and organizational inertia theories, offering new insights into how digitalization influences exploratory innovation. However, these theoretical frameworks have certain limitations when applied to artistic entrepreneurs in the creative industries. Specifically, organizational learning theory tends to focus on relational assets, overlooking the role of market sensing capabilities in driving exploratory innovation. Job demands theory primarily addresses executive workload, but does not fully explore how flexibility and autonomy in the creative industries foster innovation. Although the integrated perspective of dynamic capabilities and organizational inertia highlights a company’s ability to respond to environmental changes, it underestimates the impact of external factors such as rapid technological changes and market uncertainty on the innovation process.

Therefore, existing theoretical frameworks have not sufficiently integrated the interaction between a company’s internal capabilities and the external environment when analyzing the factors influencing exploratory innovation. In the creative industries, artistic entrepreneurs often face challenges such as resource scarcity and informality [30,31]. This study combines dynamic capabilities theory with open systems theory to analyze how artistic entrepreneurs engage in exploratory innovation in a rapidly changing environment. Dynamic capabilities theory emphasizes how organizations adjust and reconfigure their resources to respond to environmental changes, while open systems theory reveals the impact of external markets, institutions, and technologies on innovation [16–18]. By integrating these two theories, this paper will comprehensively explore the mechanisms through which artistic entrepreneurs achieve exploratory innovation in complex and dynamic environments.

### Theoretical framework

#### Dynamic capabilities: An analysis based on dynamic capabilities theory.

Since the introduction of dynamic capabilities theory, scholars have progressively expanded and refined it to address the needs of different fields and industries [32–34]. This theory is proposed to explain how organizations can gain and sustain a competitive advantage in rapidly changing environments [16]. The core idea is that organizations not only need to optimize existing resources to maintain competitiveness, but also must possess the ability to flexibly respond to environmental changes and reconfigure their resources [16,17]. Unlike traditional resource-based theory, dynamic capabilities theory emphasizes how organizations can leverage internal resource allocation, integration, and reorganization to drive innovation and transformation in uncertain and complex external environments [16,35].

Dynamic capabilities are typically categorized into three key dimensions: sensing capabilities, leveraging capabilities, and reconfiguring capabilities [16,36]. These dimensions describe how organizations update their perception of external markets, strengthen their technological application capabilities, and enhance their strategic flexibility to achieve innovation and competitive advantage. In this study, we use market sensing capability, technological capability, and strategic flexibility to represent the three core dimensions of dynamic capabilities: sensing, leveraging, and reconfiguring to analyze their impact on artistic entrepreneurs’ exploratory innovation. These three core capabilities are interdependent and together form the foundation for artistic entrepreneurs to navigate dynamic market environments and achieve exploratory innovation.

Market sensing capability is a critical competency for organizations to cope with rapidly changing market environments [37]. In the creative industries, the swift changes in market demand, consumer preferences, and technology present both opportunities and challenges for art entrepreneurs [1]. The ability to keenly identify these changes and respond promptly enables art entrepreneurs to seize potential innovative opportunities and foster exploratory innovation. Therefore, market sensing capability is not only the foundation for driving innovation but also the core competence for art entrepreneurs to maintain a competitive advantage in highly uncertain markets [38].

Technological capability plays a crucial role in driving exploratory innovation among art entrepreneurs [39]. With the rapid development of digital technologies and the emergence of new technologies such as virtual reality and artificial intelligence, significant changes have occurred in artistic creation, distribution methods, and marketing strategies [29,40]. If businesses can master and effectively utilize these technologies, they can overcome the limitations of traditional creation, expand the boundaries of their work, and inject new vitality into their creations [41]. Especially in the process of artistic creation and presentation, the flexibility of technology allows art entrepreneurs to remain competitive in an ever-evolving market.

Strategic flexibility plays an indispensable role in the process of art entrepreneurs achieving exploratory innovation [39]. In the creative industries, the continuous changes in market demand and technological advancements require art entrepreneurs to adjust their strategic direction and resource allocation in response to shifts in the external environment [42]. Strategic flexibility equips art entrepreneurs with the adaptability needed to quickly adjust their creative direction or business model in the face of sudden market changes, helping them avoid losing direction in the uncertainty of the market. Enterprises with strategic flexibility can not only respond rapidly to market changes, even under resource constraints, but also optimize resource allocation in response to these changes, maximizing the realization of innovative outcomes [43].

#### External environment: An analysis based on open systems theory.

Open systems theory was first proposed in the 1940s, aiming to emphasize the continuous interaction between organizations and their external environments [44]. The core idea of this theory is that organizations are not only influenced by external factors such as technology, market conditions, and policies but also have an active role in shaping the environment through their actions [44,45]. In an open system, organizations continuously receive external inputs (resources, information, etc.) and output results through internal transformation processes [44,46]. Therefore, the survival and development of an organization depend on its constant attention to and flexible response to changes in the external environment.

For art entrepreneurs, open systems theory provides an effective framework to understand the important role of the external environment in driving exploratory innovation. In the creative industries, which are characterized by uncertainty and competitive pressures, the innovation of art entrepreneurs is not only influenced by their internal resources and capabilities but also closely linked to technological changes, institutional frameworks, and market demands in the external environment [47,48]. Consequently, this study identifies technological turbulence, institutional pressures, and market growth as external environmental factors influencing the exploratory innovation of art entrepreneurs.

Technological turbulence is a critical external factor influencing the exploratory innovation of art entrepreneurs. The speed and depth of technological change are often accompanied by significant technological innovations and uncertainties within industries [39]. In the creative industries, the rapid development of technology forces art entrepreneurs to constantly explore new technological paths and market opportunities to remain competitive. For example, the widespread adoption of digital technologies has not only provided new forms of artistic expression but has also altered the market demand for art products, thereby driving innovation among art entrepreneurs [3,49]. Therefore, technological turbulence is a key factor that art entrepreneurs must consider when facing a constantly changing technological environment.

Institutional pressures also play a crucial role in the innovation process of art entrepreneurs. These pressures stem from government policies, laws and regulations, industry standards, and market access conditions within the cultural industry [50]. In practical business operations, organizations must not only adapt to external technological environments but also address external institutional constraints and pressures [18,51]. For example, changes in government cultural policies may prompt art entrepreneurs to alter their creative direction or business model to comply with new institutional requirements. While institutional pressures can provide a driving force for innovation, they may also impose limitations [52,53]. Therefore, institutional pressure is an external variable that cannot be overlooked, as it affects the innovative behavior of art entrepreneurs in complex policy environments.

Market growth refers to the expansion of market demand and its influence on the innovation decisions of art entrepreneurs. It is typically accompanied by changes in consumer preferences and adjustments in market structure, which directly shape the innovation strategies adopted by art entrepreneurs [39,54]. As a key element of the external environment, the market provides various forms of input to organizations, and these inputs have a profound impact on their innovation paths and strategic choices [16]. Rapid market growth pushes entrepreneurs to continuously adjust their innovation strategies to meet shifting consumer needs and respond to increasing industry competition [55]. This is particularly true in the creative industries, where art entrepreneurs often rely on changes in market demand to inspire innovation. Therefore, market growth is a critical external factor influencing the exploratory innovation of art entrepreneurs.

### Model construction

**Fig 1:**
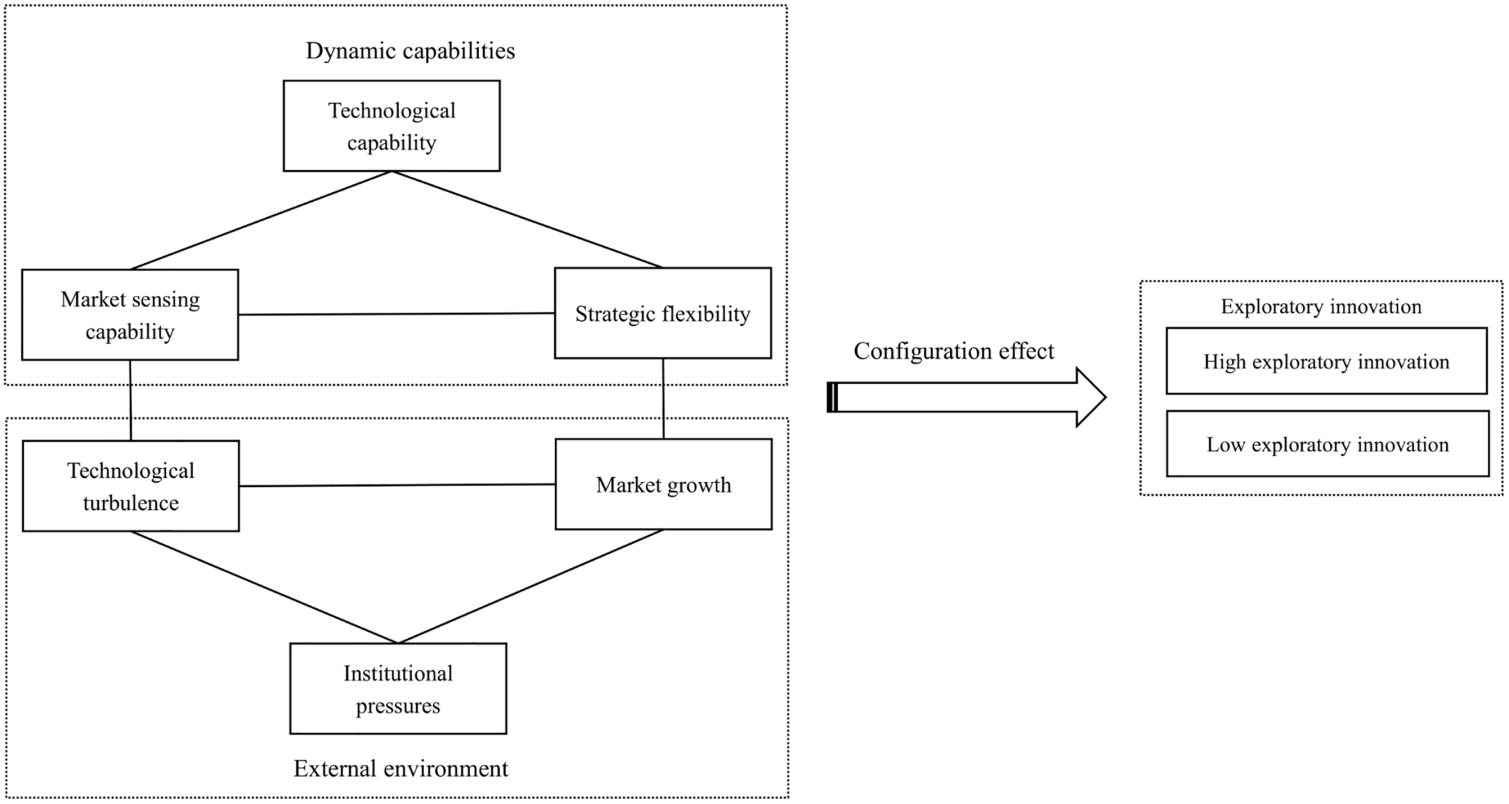
Proposed conceptual model.

The exploratory innovation of art entrepreneurs is influenced by a range of internal and external factors. This section aims to develop a conceptual model to examine how art entrepreneurs achieve exploratory innovation in complex environments. The model integrates dynamic capabilities and external environmental factors, drawing on the theories of dynamic capabilities and open systems to analyze how art entrepreneurs drive innovation amid constantly evolving market and technological landscapes. The conceptual model is illustrated in Fig 1.

## Methodology

### Data collection

The data for this study were collected from art entrepreneurs engaged in China’s cultural and creative industries, including visual arts, advertising design, theater, and multimedia art. To ensure that the measurement instruments were appropriate for this context, a self-administered questionnaire was developed based on established scales and refined through a pilot test with 15 art entrepreneurs. Feedback from the pilot was incorporated to improve clarity and enhance content validity.

In selecting participants, we adopted a purposive sampling strategy supplemented by convenience sampling. This approach was designed to maximize diversity and representativeness across different sectors of the cultural and creative industries. Data were gathered through three complementary channels: (1) Online distribution via platforms such as WeChat and QQ broadened the reach to geographically dispersed respondents. (2) Collaboration with art incubators and creative industry parks enabled access to entrepreneurs embedded in professional networks. (3) On-site distribution at cultural exhibitions and art events allowed us to target practitioners who were actively engaged in creative practice.

Participant recruitment took place from May 30 to July 30, 2025. To mitigate the risk of common method bias (CMB), several procedural controls were embedded in the questionnaire design: (1) Respondents were assured of anonymity and confidentiality to reduce evaluation apprehension. (2) The measurement of independent and dependent variables was deliberately separated: items on antecedent factors were presented in the first section, demographic and control variables were placed in the middle, and items measuring exploratory innovation (the dependent variable) were arranged in the final section. (3) Several attention check items were inserted throughout the questionnaire to identify inattentive responses and further reduce method bias. In total, 282 questionnaires were initially received. Following established quality criteria, responses were excluded if they contained substantial missing data, were completed in unrealistically short times, or displayed patterned answering behaviors. After this screening process, 203 valid questionnaires remained for analysis. The demographic characteristics of the respondents are presented in Table 1.

**Table 1:** Descriptive samples.

Category	Items	Frequency	Percentage(%)
Gender	Male	108	53.2
	Female	95	46.8
Age	18–25 years	47	23.2
	26–35 years	78	38.4
	36–45 years	50	24.6
	46 years and above	28	13.8
Education level	Associate degree or below	97	47.8
	Bachelor’s degree	76	37.4
	Postgraduate degree	30	14.8
Years of experience	0–2 years	53	26.1
	3–5 years	98	48.3
	6–10 years	36	17.7
	More than 10 years	16	7.9
Company location	First-tier city	69	34.0
	Second-tier city	65	32.0
	Third-tier city	51	25.1
	Other	18	8.9

### Ethics statement

This study was reviewed and approved by the Medical Ethics Committee of Baise University (No. BSU20250515) and the Ethics Committee of China Three Gorges University (No. GTGU20250501). All procedures were conducted in accordance with the Declaration of Helsinki and its later amendments. Written informed consent was obtained from all participants before data collection, and all data were collected anonymously to ensure confidentiality and privacy protection.

### Measures

To ensure content validity and contextual appropriateness, all constructs in the questionnaire were measured using established scales that were systematically adapted to the cultural and creative industries. A 7-point Likert scale was used for all items. The adaptation process followed a structured approach: (1) retaining the original construct structure and measurement logic of each scale, (2) rewriting item wording to align with the operational and creative characteristics of art enterprises, and (3) conducting expert review and a pilot test to refine semantic clarity.

Strategic flexibility, technological capability, technological turbulence, and market growth were adapted from Zhou and Wu [39]. These items were originally developed for manufacturing and technology-intensive firms; therefore, they were semantically transformed to reflect flexibility in artistic production, digital creation, resource allocation, and the dynamics of cultural markets. Market sensing capability was measured using the framework developed by Kohli et al. [56]. Given the unique nature of consumer interaction and trend formation in the creative industries, the items were adapted to emphasize audience engagement, informal information-gathering at art fairs or exhibitions, and sensing shifts in cultural or aesthetic trends. Institutional pressures were assessed using the scale from Tang et al. [50]. Because art enterprises face specific regulatory environments related to cultural policy, intellectual property, and content governance, item wording was modified to reflect compliance with cultural industry standards and IP regulations. The exploratory innovation scale was developed on the basis of the exploratory orientation scale proposed by Lubatkin et al. [57], retaining its original construct dimensions and measurement logic. Subsequently, drawing on Jansen et al.’s [24] conceptualization of exploratory innovation, the items were semantically adapted to capture innovation activities related to new products, creative forms, and new markets within the context of art enterprises.

### Data analysis strategy

Exploratory innovation in the context of art entrepreneurship is shaped by complex and often non-linear causal mechanisms. To capture this complexity, the present study employs a mixed-method approach that combines fsQCA with NCA. FsQCA is particularly effective in examining how different configurations of antecedent conditions can jointly lead to the same outcome, highlighting the principles of equifinality and asymmetry [21]. This allows the analysis to move beyond single-variable effects and reveal multiple, equally valid pathways to exploratory innovation. Nevertheless, the necessity tests within fsQCA rely heavily on qualitative assessment, which may lead to an underestimation of truly indispensable conditions [19]. The NCA provides a quantitative evaluation of necessity, thereby enabling a more precise identification of conditions that function as critical thresholds for innovation. By integrating the configurational insights of fsQCA with the quantitative rigor of NCA, this strategy offers a more comprehensive and reliable account of how various factors constrain or enable the exploratory innovation of art entrepreneurs [19].

## Results

### CMB test

Since self-reported questionnaires were the sole method of data collection, there was a potential risk of CMB. To address this concern, this study first conducted Harman’s single-factor test, where all items were loaded into an exploratory factor analysis with the number of factors constrained to one. The results showed that the single factor accounted for only 35.1% of the total variance [58]. In addition, variance inflation factor (VIF) values were examined within the research model, and all were well below the commonly accepted threshold, ranging from 1.022 to 1.507, indicating no multicollinearity concerns [59]. These results suggest that CMB was not a serious issue.

### Exploratory factor analysis

The results of the exploratory factor analysis (EFA) are presented in Table 2. The Kaiser–Meyer–Olkin (KMO) value is 0.906 and Bartlett’s test of sphericity is significant (p < 0.001), indicating that the data are suitable for factor analysis. Principal component analysis extracted seven factors with eigenvalues greater than 1, explaining 70.356% of the total variance. Overall, the measurement items load strongly on their corresponding constructs with relatively low cross-loadings. Although a few items exhibit relatively lower loadings, they remain within the acceptable threshold. These results suggest that the measurement scales demonstrate satisfactory convergent and discriminant validity.

**Table 2:** Results of exploratory factor analysis.

Constructs	Items	Components						
		1	2	3	4	5	6	7
Market sensing capability(MSC)	MSC1	**0.745**	0.156	0.188	0.151	0.063	0.025	0.284
	MSC2	**0.777**	0.108	0.177	0.089	0.156	−0.034	0.288
	MSC3	**0.740**	0.097	0.081	−0.025	0.047	−0.055	0.192
	MSC4	**0.801**	0.037	0.158	0.029	0.107	0.054	0.059
	MSC5	**0.794**	0.042	0.148	0.044	0.174	−0.007	0.071
	MSC6	**0.748**	0.154	0.090	0.138	0.107	0.049	0.002
	MSC7	**0.749**	0.083	0.028	0.157	0.079	0.000	−0.063
	MSC8	**0.685**	0.155	0.159	0.151	0.097	0.058	0.019
	MSC9	**0.750**	0.130	0.166	0.136	0.044	−0.014	−0.079
	MSC10	**0.753**	0.118	0.216	0.143	0.130	0.008	0.181
Technological capability(TC)	TC1	0.158	0.138	0.193	**0.752**	0.153	0.052	0.083
	TC2	0.105	0.114	0.115	**0.743**	0.108	−0.033	0.257
	TC3	0.085	0.139	0.196	**0.774**	0.147	0.061	0.149
	TC4	0.143	0.153	0.217	**0.659**	0.063	0.031	0.047
	TC5	0.207	0.227	0.168	**0.714**	0.149	−0.005	−0.008
Strategic flexibility(SF)	SF1	0.207	**0.753**	0.037	0.159	0.108	0.003	0.142
	SF2	0.142	**0.822**	0.113	0.151	0.110	−0.110	0.061
	SF3	0.129	**0.816**	0.122	0.137	0.157	−0.073	0.028
	SF4	0.136	**0.744**	0.203	0.025	0.055	0.129	0.093
	SF5	0.139	**0.689**	0.252	0.160	0.025	−0.046	0.094
	SF6	0.049	**0.714**	0.129	0.159	0.120	−0.107	0.101
Technological turbulence(TT)	TT1	0.142	0.164	0.281	0.159	**0.776**	0.043	0.076
	TT2	0.193	0.124	0.208	0.144	**0.843**	0.037	0.054
	TT3	0.163	0.144	0.198	0.168	**0.849**	0.021	0.098
	TT4	0.206	0.121	0.193	0.134	**0.814**	0.038	0.055
Institutional pressures(IP)	IP1	0.046	−0.067	−0.109	−0.004	0.026	**0.883**	−0.012
	IP2	0.037	−0.035	−0.085	0.032	0.022	**0.901**	0.027
	IP3	−0.020	−0.053	−0.016	0.052	0.050	**0.916**	−0.007
Market growth(MG)	MG1	0.153	0.198	0.067	0.246	0.021	−0.030	**0.825**
	MG2	0.205	0.166	0.217	0.211	0.111	0.074	**0.794**
	MG3	0.189	0.123	0.351	0.034	0.186	−0.030	**0.503**
Exploratory innovation(EI)	EI1	0.325	0.344	**0.645**	0.299	0.259	−0.062	0.025
	EI2	0.277	0.240	**0.748**	0.224	0.193	−0.044	0.084
	EI3	0.290	0.164	**0.731**	0.192	0.210	−0.043	0.127
	EI4	0.201	0.189	**0.770**	0.196	0.191	−0.047	0.148
	EI5	0.176	0.209	**0.734**	0.260	0.224	−0.100	0.135
	EI6	0.254	0.108	**0.565**	0.234	0.254	−0.135	0.186

KMO=0.906, df=666, p-value<0.001, Cumulative Variance Explained=70.4%.

### Measurement model assessment

The results of the measurement model evaluation are presented in Table 3, indicating that all latent variables used in the study exhibit strong reliability and convergent validity. Firstly, all standardized factor loadings exceed 0.70, indicating that the observed variables effectively reflect their corresponding constructs [59]. In terms of reliability, all constructs have Cronbach’s α above 0.70 and composite reliability (CR) values exceeding 0.80, meeting the commonly recommended threshold of 0.70, which indicates a high level of internal consistency [59]. Furthermore, the average variance extracted (AVE) for each construct is greater than 0.632, surpassing the 0.50 benchmark and supporting the convergent validity of the measurement model [59,60].

**Table 3:** Measurement model results.

Constructs	Items	Loadings
Exploratory innovation(EI) (Cronbach’s α = 0.925;CR = 0.942;AVE = 0.726)	EI1:We frequently develop novel artistic products or creative services that extend beyond our existing scope.	0.892
	EI2:We experiment with new artistic forms, creative technologies, or service concepts to explore emerging possibilities.	0.892
	EI3:We actively pursue opportunities in new cultural or artistic markets and engage new audience or customer groups.	0.848
	EI4:We adopt new distribution channels or digital platforms to disseminate, exhibit, or promote our artistic products or services.	0.861
	EI5:We commercialize artistic products or services that are entirely new to our organization.	0.865
	EI6:We are willing to accept creative demands that exceed our current artistic–market boundaries and seek breakthrough innovations.	0.761
Institutional pressures(IP) (Cronbach’s α = 0.894;CR = 0.932;AVE = 0.820)	IP1:We innovate to meet the standards or regulatory requirements in the cultural and creative industries.	0.926
	IP2:We innovate to avoid infringement on legislation, regulations, and standards.	0.899
	IP3:We innovate to ensure compliance with intellectual property laws and regulations.	0.891
Market growth(MG) (Cronbach’s α = 0.770;CR = 0.867;AVE = 0.685)	MG1:The growth rate of this industry over the past three years is very high.	0.832
	MG2:Market demand in this industry is growing rapidly.	0.887
	MG3:There are many potential customers in this industry providing mass-market opportunities.	0.759
Market sensing capability(MSC) (Cronbach’s α = 0.935;CR = 0.945;AVE = 0.63)	MSC1:We regularly meet with clients, audiences, or collaborators to understand their future needs and artistic preferences.	0.837
	MSC2:Members from creative or production teams interact directly with users to learn how to improve artistic offerings.	0.846
	MSC3:We frequently conduct internal or informal research to stay updated on cultural or creative trends.	0.865
	MSC4:We are slow to perceive changes in audience tastes and expectations. (R)	0.752
	MSC5:We regularly collect feedback from end users to evaluate the quality and resonance of our artistic products or services.	0.805
	MSC6:We often engage with key influencers (e.g., curators, media, organizers) to understand audience behavior.	0.814
	MSC7:We gather industry intelligence through informal conversations at art fairs, exhibitions, or peer gatherings.	0.766
	MSC8:Insights about competitors or peer artists are shared across different departments.	0.732
	MSC9:We are slow to detect major shifts in the creative industry (e.g., policy or platform changes). (R)	0.743
	MSC10:We routinely evaluate how external factors (e.g., regulations, cultural shifts) may affect our audiences.	0.776
Strategic flexibility(SF) (Cronbach’s α = 0.891;CR = 0.917;AVE = 0.648)	SF1:We flexibly allocate marketing resources (e.g., promotion, exhibition, distribution) to support different types of artistic products or creative projects.	0.796
	SF2:We flexibly adjust creative resources (e.g., production teams, technical support) to develop diverse artistic works or service formats.	0.861
	SF3:We maintain high flexibility in the design and presentation of artistic works to support a wide range of possible applications.	0.853
	SF4:We redefine our artistic product strategies and target segments in response to audience preferences or cultural trends.	0.772
	SF5:We reconfigure the chains of resources used for creating, producing, and delivering artistic outputs to fit new creative directions.	0.781
	SF6:We redeploy organizational resources effectively to support new artistic projects or innovation strategies.	0.763
Technological capability(TC) (Cronbach’s α = 0.895;CR = 0.899;AVE = 0.640)	TC1:Acquiring important technological information relevant to artistic creation or digital media.	0.835
	TC2:Identifying new technology opportunities.	0.785
	TC3:Responding to technological changes.	0.83
	TC4:Mastering state-of-the-art technologies (e.g., VR, AI-assisted creation).	0.739
	TC5:Constantly developing a series of technological or creative innovations.	0.807
Technological turbulence(TT) (Cronbach’s α = 0.919;CR = 0.943;AVE = 0.804)	TT1:The technology in this industry is changing rapidly.	0.885
	TT2:Technological changes provide substantial opportunities in this industry.	0.912
	TT3:A large number of new artistic ideas have been enabled by technological breakthroughs.	0.916
	TT4:It is very difficult to forecast where the relevant technology will be in the next few years.	0.874

The results of discriminant validity are shown in Table 4. In the Fornell-Larcker criterion, the square root AVE (diagonal values) of each latent variable was higher than the correlation coefficients between it and the other variables, indicating that the constructs were well differentiated from each other [60]. The Heterotrait-Monotrait(HTMT) ratios further validated this result, with all pairs of constructs with each other having HTMT values below the commonly used threshold of 0.85, with a maximum value of 0.653, which is well below the warning line, indicating that there are no potential discriminant validity issues [61].

**Table 4:** Discriminant validity results.

Constructs	Model discriminant validity						
	1	2	3	4	5	6	7
1.EI	**0.854**	0.144	0.585	0.614	0.571	0.653	0.636
2.IP	−0.138	**0.906**	0.044	0.065	0.127	0.052	0.056
3.MSC	0.553	0.023	**0.795**	0.499	0.401	0.428	0.430
4.MG	0.526	−0.014	0.437	**0.828**	0.484	0.548	0.419
5.SF	0.524	−0.100	0.370	0.399	**0.805**	0.495	0.402
6.TC	0.584	0.027	0.389	0.442	0.434	**0.800**	0.479
7.TT	0.588	0.045	0.403	0.363	0.364	0.428	**0.897**

$\text{Diagonal }=\;\sqrt{\text{AVE}}$; Below diagonal: estimated correlations; Above diagonal: HTMT coefficient of correlations, EI Exploratory innovation, IP Institutional pressures, MG Market growth, MSC Market sensing capability, SF Strategic flexibility, TC Technological capability, TT Technological turbulence.

### Data calibration

Grounded in the theoretical foundations of set-theoretic methods and informed by the empirical distribution of the observed data, we calibrated all condition variables using Ragin’s [62] direct method. Consistent with established practice for seven-point Likert measures, the three anchor points were defined at the 95th percentile, the 50th percentile, and the 5th percentile. Because fuzzy-set intersection rules treat values of 0.5 as logically ambiguous, we followed Ragin [62] and [63] by adding a small constant (0.001) to any calibrated value equal to 0.5 to avoid indeterminacy during truth table construction. Calibration anchors and descriptive statistics for all constructs are presented in Table 5.

**Table 5:** Calibration thresholds and descriptive statistics of study constructs.

Constructs	Full membership	Crossover point	Full-non-membership	Skewness	Kurtosis
1.EI	6.167	4.333	1.333	−0.395	−1.206
2. IP	6.667	5.000	1.667	−0.559	−0.896
3.MG	6.600	4.333	1.667	−0.028	−1.026
4.MSC	6.000	4.800	1.700	−0.670	−0.868
5. SF	5.667	4.333	1.500	−0.499	−0.958
6. TC	5.600	4.200	1.200	−0.481	−0.979
7.TT	6.500	4.000	1.250	−0.151	−0.977

*EI* Exploratory innovation, *IP* Institutional pressures, *MG* Market growth, *MSC* Market sensing capability, *SF* Strategic flexibility, *TC* Technological capability, *TT* Technological turbulence.

### NCA results

To examine whether any variables constitute necessary conditions for the outcome, we conducted an NCA using both ceiling regression (CR) and ceiling envelopment (CE) methods. Following Dul et al. [64], a condition can be evaluated as necessary when its effect size (d) exceeds the recommended threshold of 0.1, and the permutation test yields a significant result (p < 0.01). Given its non-parametric nature and robustness to non-linear boundaries, CE results were used as the primary basis for interpretation [65]. As shown in Table 6, although several variables, such as market sensing capability, technological capability, strategic flexibility, technological turbulence, and market growth, exhibit statistically significant p-values under CE, none of them achieve the required effect size threshold. Therefore, based on the CE analysis, no variable meets the necessary condition criteria.

**Table 6:** Analysis of the results of the necessary conditions of the NCA method.

Condition	Method	Accuracy	Ceiling zone	Effect size(d)	p values
MSC	CR	94.1%	0.087	0.098	0.134
	CE	100%	0.025	0.028	0.004
TC	CR	83.7%	0.139	0.156	0.016
	CE	100%	0.038	0.043	0.000
SF	CR	98.5%	0.017	0.019	0.186
	CE	100%	0.020	0.022	0.002
TT	CR	93.1%	0.100	0.115	0.017
	CE	100%	0.037	0.043	0.000
IP	CR	90.1%	0.043	0.048	0.192
	CE	100%	0.019	0.021	0.057
MG	CR	80.2%	0.174	0.194	0.011
	CE	100%	0.043	0.048	0.000

*EI* Exploratory innovation, *IP* Institutional pressures, *MG* Market growth, *MSC* Market sensing capability, *SF* Strategic flexibility, *TC* Technological capability, *TT* Technological turbulence.

To further examine the constraining effects of condition variables at different outcome levels, this study conducts a bottleneck level analysis using the NCA method as a supplementary validation. As shown in Table 7, when the outcome level reaches 90% or above, technological capability, market growth, and technological turbulence exhibit clear bottleneck characteristics. At the 100% outcome level, the minimum required values for technological capability, market growth, and institutional pressure are 58.6%, 84.2%, and 82.4%, respectively. In contrast, other variables show relatively weak constraining effects. Although this analysis does not involve significance testing [65], it serves to identify key limiting factors in achieving high-performance outcomes.

**Table 7:** Analysis results of the NCA method bottleneck level (100%).

EI	MSC	TC	SF	TT	IP	MG
0	NN	NN	NN	NN	NN	NN
10	NN	NN	NN	NN	NN	NN
20	NN	NN	NN	NN	NN	NN
30	NN	NN	0	NN	NN	NN
40	NN	NN	0.8	NN	NN	NN
50	NN	3.6	1.6	NN	NN	NN
60	7.6	14.6	2.3	0.7	NN	11.3
70	15.6	25.6	3.1	14.7	NN	29.5
80	23.6	36.6	3.9	28.7	NN	47.7
90	31.6	47.6	4.7	42.7	12.1	66
100	39.6	58.6	5.4	56.7	82.4	84.2

The bottleneck level (%) analysis result is calculated using the CR method, *EI* Exploratory innovation, *IP* Institutional pressures, *MG* Market growth, *MSC* Market sensing capability, *SF* Strategic flexibility, *TC* Technological capability, *TT* Technological turbulence.

To further assess the necessity of individual conditions for the outcome variable, this study applies the fsQCA method. A condition can be preliminarily considered a “necessary condition” if its consistency score exceeds 0.9. As shown in Table 8, none of the condition variables or their negations surpass the 0.9 consistency threshold under either high or low levels of exploratory innovation. This indicates that no single condition can be regarded as a strictly necessary condition for the outcome.

**Table 8:** fsQCA analysis results of necessary conditions.

Condition	High-level EI		Low-level EI	
	Consistency	Coverage	Consistency	Coverage
MSC	0.743	0.744	0.535	0.521
~MSC	0.522	0.536	0.737	0.736
TC	0.737	0.753	0.525	0.521
~TC	0.531	0.535	0.751	0.735
SF	0.718	0.763	0.513	0.530
~SF	0.557	0.541	0.770	0.726
TT	0.781	0.773	0.517	0.497
~TT	0.491	0.512	0.764	0.772
IP	0.604	0.580	0.690	0.644
~IP	0.629	0.676	0.550	0.575
MG	0.700	0.769	0.495	0.528
~MG	0.570	0.537	0.783	0.717

“~” is used to represent the lack of a condition, *EI* Exploratory innovation, *IP* Institutional pressures, *MG* Market growth, *MSC* Market sensing capability, *SF* Strategic flexibility, *TC* Technological capability, *TT* Technological turbulence.

### Analysis of sufficient configurations

The analysis of configurational sufficiency aims to examine whether different combinations of antecedent conditions are sufficient for producing a specific outcome. Following the approach of Fiss [20], this study sets the raw consistency threshold at 0.8, the PRI consistency threshold at 0.8, and the minimum case frequency at 3. Using the fsQCA 4.0 software, three types of solutions can be generated: simple, intermediate, and complex. This study primarily relies on the intermediate solution, supplemented by the simple solution. Conditions that appear in both the intermediate and simple solutions are defined as “core conditions” [20].

As shown in Table 9, six configurations lead to high exploratory innovation, with a solution consistency of 0.923 and coverage of 0.625. In contrast, there is one configuration associated with low exploratory innovation, with a solution consistency of 0.929 and coverage of 0.391. Both the consistency and coverage values exceed the critical thresholds, indicating that the empirical findings are robust. Furthermore, the consistency of both individual and overall solutions is above 0.8, suggesting that the configurations can be regarded as credible sufficient conditions for the outcome variable.

**Table 9:** High-level and low-level EI configurations.

Causal condition	High-level EI						Low-level EI
	H1		H2		H3		L1
	H1a	H1b	H2a	H2b	H3a	H3b	L1a
MSC	●			⨂	●	●	⨂
TC	●	●	⨂	●	●	⨂	⨂
SF	●	●	●	⨂	⨂	●	⨂
TT		●	●	●	●	●	⨂
IP		⨂	⨂	⨂	●	●	
MG	●	⨂	●	●	⨂	⨂	⨂
Raw coverage	0.441	0.283	0.274	0.221	0.255	0.224	0.391
Unique coverage	0.135	0.036	0.033	0.012	0.040	0.022	0.391
Consistency	0.946	0.958	0.968	0.957	0.946	0.963	0.929
Solution consistency	0.923						0.929
Solution coverage	0.625						0.391

Core conditions are indicated by ● (presence) and ⨂ (absence); peripheral conditions are presented by ● (presence) and ⨂ (absence); when causal conditions are either present or absent, they are presented by blank spaces, *EI* Exploratory innovation, *IP* Institutional pressures, *MG* Market growth, *MSC* Market sensing capability, *SF* Strategic flexibility, *TC* Technological capability, *TT* Technological turbulence.

To enhance theoretical coherence and structural clarity, this study follows the commonly used principle of “mechanism-based categorization” in fsQCA research. Specifically, paths with similar logical structures and core condition compositions are merged: H1a and H1b are combined into Path H1, H2a and H2b into Path H2, and H3a and H3b into Path H3. Each of these consolidated paths represents a distinct and representative mechanism underlying the formation of exploratory innovation.

Path H1 (a combination of H1a and H1b) can be characterized as “endogenous capability-driven”. It is defined by the core presence of technological capability and strategic flexibility, which together form a critical internal foundation for driving exploratory innovation. This suggests that when art entrepreneurs possess strong technological competence and agile strategic adjustment capabilities, they can achieve breakthrough innovations through internal resource reconfiguration—even in the absence of highly dynamic external environments. This path highlights the synergistic effect among internal dynamic capabilities and underscores the direct driving role of organizational capabilities in fostering innovation.

Path H2 (a merger of H2a and H2b) can be summarized as “externally opportunity-driven”, characterized by the core presence of technological turbulence and market growth, alongside the core absence of institutional pressure. This indicates that under conditions of rapid technological change and expanding markets, firms are more inclined toward exploratory innovation if they operate in relatively relaxed institutional environments. This configuration emphasizes that environmental uncertainty and growth create space for experimentation and breakthroughs, while reduced institutional constraints help alleviate path dependence and behavioral rigidity.

Path H3 (formed by combining H3a and H3b) can be defined as “perception–response driven”. It is marked by the core presence of market sensing capability and technological turbulence, and the core absence of market growth. This suggests that even in contexts where market expansion is limited, firms with strong market insight and the ability to respond swiftly to technological changes can still identify and seize new exploratory opportunities. This path reflects a cognitive–behavioral mechanism in which perception leads to action, enabling breakthrough innovation under the pressure of technological transformation.

In contrast, the only configuration leading to low levels of exploratory innovation, Path L1a (labeled “resource–environment deficiency”), is characterized by a dual lack of internal capabilities and external environmental stimuli. In this configuration, market sensing capability, strategic flexibility, and institutional pressure are all core absent conditions, while technological turbulence and market growth are marginally absent. This result indicates that when a firm lacks both the internal capacity for resource coordination and cognitive responsiveness and operates in a stable external environment with limited change or pressure, its level of exploratory innovation is significantly diminished. This path reflects a systemic lack of innovation, dynamism and represents a typical low-performance configuration.

### Robustness tests

To verify the robustness of the configurational conditions for exploratory innovation among art entrepreneurs, this study employed two validation strategies. First, based on the original baseline model, the PRI consistency threshold was increased from 0.80 to 0.85. The adjusted results remained consistent with the baseline configurations, indicating that the findings are robust under different threshold settings. Second, the case frequency threshold was raised from 3 to 4. The resulting configurations demonstrated a clear subset relationship with those of the baseline model, further confirming the stability of the research conclusions. These robustness checks provide strong support for the explanatory power and theoretical reliability of the causal configurations identified in this study.

## Discussion

### Summary of findings

This study focuses on art entrepreneurs and integrates dynamic capabilities theory with open systems theory to construct a model explaining the mechanisms influencing exploratory innovation. By employing a mixed-methods approach that combines NCA and fsQCA, the research systematically examines the necessity and sufficiency of six antecedent conditions in driving exploratory innovation. The goal is to uncover the complex causes and diverse pathways that lead to exploratory innovation in the creative industries.

In the NCA analysis, no single variable emerges as a necessary condition for exploratory innovation. In the sufficiency analysis, fsQCA identifies three distinct configurations that lead to high levels of exploratory innovation, each representing different driving logics: internal capability development, external environmental stimuli, and perception-response mechanisms. Path H1 emphasizes the synergy between technological capability and strategic flexibility, forming an “internally driven capability path.” This suggests that firms can achieve breakthrough innovation by leveraging internal resource advantages. Path H2 highlights the dynamic interplay of technological turbulence and market growth, coupled with the absence of institutional pressure, shaping an “externally stimulated opportunity path.” This configuration underscores the positive influence of external opportunities and relaxed institutional constraints on innovation. Path H3 focuses on the interaction between market sensing capability and technological turbulence. In the absence of market growth, this “perception-response driven path” indicates that even in demand-deficient markets, firms can identify and create exploratory opportunities through heightened environmental awareness and technological adaptability. Additionally, the study identifies a negative configuration path associated with low levels of exploratory innovation, characterized by a lack of internal dynamic capabilities and insufficient external stimulation.

### Theoretical implications

This study explores the formation mechanism of exploratory innovation among creative entrepreneurial firms from a configurational perspective, providing several important insights for related theoretical research.

Firstly, this study deepens the understanding of the formation mechanism of exploratory innovation from the perspective of configurational logic. By employing the fsQCA method, three configurational paths leading to high levels of exploratory innovation are identified, indicating that exploratory innovation is not driven by a single factor but rather emerges from different combinations of internal capabilities and external environmental conditions. This finding not only reveals the equifinality and multiple pathways underlying the formation of exploratory innovation but also provides a new analytical framework for understanding complex innovation behaviors [14,22]. Compared with studies that focus solely on the net effects of individual variables, this study emphasizes the synergistic interactions among multiple conditions, thereby responding to the argument in organizational research that theoretical explanations should clarify the causal logic underlying observed phenomena [14,66].

Secondly, this study extends the application of dynamic capabilities theory in the context of creative entrepreneurship. Specifically, market sensing capability, technological capability, and strategic flexibility are conceptualized as key capability dimensions, and the findings reveal that these capabilities form multiple innovation pathways under different environmental conditions. This suggests that dynamic capabilities do not operate in isolation but jointly facilitate exploratory innovation through various capability configurations. The results not only enrich the application of dynamic capabilities theory in the study of creative industries but also highlight the importance of capability configuration in explaining firm innovation behavior [32,33]. Following the fundamental logic of theoretical contribution, this study clarifies the key factors involved (what), the relationships among these factors (how), and the underlying mechanisms through which these relationships influence outcomes (why), thereby advancing the development of related theories [67].

Finally, from the perspective of open systems theory, this study reveals the interactive relationship between external environmental conditions and firm capabilities. The findings indicate that external environmental factors such as technological turbulence, market growth, and institutional pressures do not exert a single or stable influence on exploratory innovation. Instead, their effects emerge through different combinations with internal capability structures. For instance, in environments characterized by technological turbulence, strong market sensing capability helps firms identify new innovation opportunities, while strategic flexibility enables them to rapidly adjust resource allocation to achieve exploratory innovation [37,43]. This suggests that exploratory innovation exhibits strong contextual dependence, and its formation mechanism depends on the alignment between firms’ capability structures and external environmental conditions. Consequently, this study not only deepens the application of open systems theory in innovation research but also responds to recent theoretical developments emphasizing the importance of contextualized explanations, highlighting that theories need to be continuously refined and developed within specific contexts [68].

### Practical implications

The findings of this study provide several practical implications for creative entrepreneurial firms and policymakers seeking to foster exploratory innovation.

First, at the organizational level, managers should adopt a systemic approach to developing innovation capabilities rather than focusing on the enhancement of a single capability in isolation. Exploratory innovation typically relies on the coordinated interaction of multiple capabilities. Therefore, firms should emphasize the integration and alignment of key capabilities such as market sensing, technological development, and strategic flexibility. For example, establishing cross-functional collaboration mechanisms can help integrate market information acquisition, technological development, and strategic decision-making processes, thereby forming a more coherent innovation capability structure. Moreover, during the resource-constrained entrepreneurial stage, managers may prioritize investments in capabilities that simultaneously enhance opportunity recognition and resource integration efficiency in order to sustain exploratory innovation.

Second, at the strategic level, firms should establish mechanisms that enable the continuous identification and exploitation of innovation opportunities. In rapidly changing technological and market environments, exploratory innovation often depends on timely opportunity recognition and strategic responsiveness. Firms can achieve this by regularly monitoring technological trends, shifts in market demand, and changes in competitive dynamics, and by supporting innovation initiatives through flexible resource allocation and adaptive project management. Additionally, engaging in open innovation, cross-industry collaboration, and innovation networks can broaden access to external knowledge and resources.

Third, at the institutional level, policymakers should support the innovation activities of creative entrepreneurial firms by optimizing the institutional environment and strengthening the innovation ecosystem. Exploratory innovation is characterized by high uncertainty and substantial resource requirements, relying solely on internal firm capabilities is often insufficient to sustain innovation. Governments and related institutions can improve the innovation environment through various policy instruments, such as providing R&D support, establishing creative industry incubation platforms, and promoting collaboration between firms, universities, and research institutions. Furthermore, initiatives that foster creative industry clusters and strengthen innovation resource-sharing platforms can help reduce firms’ innovation costs and facilitate knowledge exchange, thereby creating a more supportive institutional environment for creative entrepreneurial firms to pursue exploratory innovation.

### Limitations and future research

Although this study provides important insights into exploratory innovation in creative entrepreneurship, several limitations should be acknowledged, which also suggest promising directions for future research.

First, this study adopts a cross-sectional research design, which limits the ability to capture the dynamic evolution of exploratory innovation over time. Exploratory innovation is often a long-term and iterative process, in which firms continuously adjust their capabilities and strategies in response to environmental changes. Future research could therefore employ longitudinal designs to examine how capability configurations evolve during different stages of entrepreneurial development. Such an approach would provide deeper insights into the dynamic mechanisms through which firms adapt their innovation strategies over time.

Second, this study focuses on creative entrepreneurship within a specific institutional and industrial context. While this context provides a valuable setting for understanding exploratory innovation among art entrepreneurs, the findings may not fully reflect innovation processes in other industries or institutional environments. Future research could explore whether similar capability–environment configurations emerge in other sectors, such as technology startups or cultural industries in different countries. Comparative studies across different contexts would help identify which innovation mechanisms are context-specific and which are more generalizable.

Third, although this study examines several key capability and environmental factors, exploratory innovation may also be influenced by additional mechanisms that were not considered in the present research. For example, individual-level factors such as entrepreneurial cognition, creativity, and risk perception may shape how entrepreneurs interpret environmental signals and make innovation decisions. Future studies could integrate micro-level psychological or cognitive factors with organizational capabilities to develop a more comprehensive understanding of exploratory innovation.

## Supporting information

S1 File Measurement scales. (DOCX)

S2 File Cleaned anonymized survey dataset. (XLSX)

S3 File Calibrated dataset for fsQCA and NCA analyses. (CSV)

S4 File NCA code. (DOCX)
