# Supplementary material for: Capability–environment configurations driving exploratory innovation in art entrepreneurs: A mixed-methods configurational study
Source: PLoS One. 2026 May 4;21(5):e0348315. doi: 10.1371/journal.pone.0348315 (PMC13138646; doi:10.1371/journal.pone.0348315)
Supplement: S1 File — (DOCX) [file pone.0348315.s001.docx]

**This study measures scales.**

| **Variable** | **Item** | **Source** |
| --- | --- | --- |
| Please indicate the extent to which the following statements reflect the practices of your art enterprise. (1 = strongly disagree, 7 = strongly agree) | | |
| Strategic flexibility  (SF) | 1.We flexibly allocate marketing resources (e.g., promotion, exhibition, distribution) to support different types of artistic products or creative projects. | Zhou & Wu(2010) |
|  | 2. We flexibly adjust creative resources (e.g., production teams, technical support) to develop diverse artistic works or service formats. |  |
|  | 3. We maintain high flexibility in the design and presentation of artistic works to support a wide range of possible applications. |  |
|  | 4. We redefine our artistic product strategies and target segments in response to audience preferences or cultural trends. |  |
|  | 5. We reconfigure the chains of resources used for creating, producing, and delivering artistic outputs to fit new creative directions. |  |
|  | 6. We redeploy organizational resources effectively to support new artistic projects or innovation strategies. |  |
| Compared to your major competitors, how would you evaluate your firm’s capabilities in the following areas? (1 = much worse, 7 = much better) | | |
| Technological capability  (TC) | 1. Acquiring important technological information relevant to artistic creation or digital media. | Zhou & Wu(2010) |
|  | 2. Identifying new technology opportunities. |  |
|  | 3. Responding to technological changes. |  |
|  | 4. Mastering state-of-the-art technologies (e.g., VR, AI-assisted creation). |  |
|  | 5. Constantly developing a series of technological or creative innovations. |  |
| Please indicate the extent to which each statement reflects the current practices of your enterprise. (1 = strongly disagree, 7 = strongly agree) | | |
| Market sensing capability  (MSC) | 1.We regularly meet with clients, audiences, or collaborators to understand their future needs and artistic preferences. | Kohli et al.(1993) |
|  | 2.Members from creative or production teams interact directly with users to learn how to improve artistic offerings. |  |
|  | 3.We frequently conduct internal or informal research to stay updated on cultural or creative trends. |  |
|  | 4.We are slow to perceive changes in audience tastes and expectations. (R) |  |
|  | 5.We regularly collect feedback from end users to evaluate the quality and resonance of our artistic products or services. |  |
|  | 6.We often engage with key influencers (e.g., curators, media, organizers) to understand audience behavior. |  |
|  | 7.We gather industry intelligence through informal conversations at art fairs, exhibitions, or peer gatherings. |  |
|  | 8.Insights about competitors or peer artists are shared across different departments. |  |
|  | 9.We are slow to detect major shifts in the creative industry (e.g., policy or platform changes). (R) |  |
|  | 10.We routinely evaluate how external factors (e.g., regulations, cultural shifts) may affect our audiences. |  |
| Please indicate the extent to which each statement reflects the current artistic or creative industry. (1 = strongly disagree, 7 = strongly agree) | | |
| Technological turbulence | 1.The technology in this industry is changing rapidly. | Zhou & Wu(2010) |
|  | 2.Technological changes provide substantial opportunities in this industry. |  |
|  | 3.A large number of new artistic ideas have been enabled by technological breakthroughs. |  |
|  | 4.It is very difficult to forecast where the relevant technology will be in the next few years. |  |
| Please indicate the extent to which each statement reflects the current artistic or creative industry. | | |
| Market growth | 1.The growth rate of this industry over the past three years is very high. | Zhou & Wu(2010) |
|  | 2.Market demand in this industry is growing rapidly. |  |
|  | 3.There are many potential customers in this industry providing mass-market opportunities. |  |
| Please indicate the extent to which each statement reflects your enterprise. | | |
| Institutional pressures | 1.We innovate to meet the standards or regulatory requirements in the cultural and creative industries. | Tang et al.(2019) |
|  | 2.We innovate to avoid infringement on legislation, regulations, and standards. |  |
|  | 3.We innovate to ensure compliance with intellectual property laws and regulations. |  |
| Please indicate the extent to which each statement reflects your art enterprise. | | |
| Exploratory Innovation | 1.We frequently develop novel artistic products or creative services that extend beyond our existing scope. | Lubatkin et al.(2006); Jansen et al.(2006) |
|  | 2.We experiment with new artistic forms, creative technologies, or service concepts to explore emerging possibilities. |  |
|  | 3.We actively pursue opportunities in new cultural or artistic markets and engage new audience or customer groups. |  |
|  | 4.We adopt new distribution channels or digital platforms to disseminate, exhibit, or promote our artistic products or services. |  |
|  | 5.We commercialize artistic products or services that are entirely new to our organization. |  |
|  | 6.We are willing to accept creative demands that exceed our current artistic–market boundaries and seek breakthrough innovations. |  |
| Attention test question | To show you are paying attention, please select “Strongly Disagree” for this statement. Scale: 1 = Strongly Disagree, 7 = Strongly Agree. | |
| Demographic Characteristics | | |
| 1.Gender: Male□ Female□ | | |
| 2.Age: 18–25 years □ 26–35 years□ 36–45 years□ 46 years and above□ | | |
| 3.Education Level: Associate degree or below□ Bachelor's degree□ Postgraduate degree□ | | |
| 4.Years in Art Entrepreneurship: 0–2 years □ 3–5 years□ 6–10 years□ More than 10 years□ | | |
| 5.Company Location: First-tier city□ Second-tier city□ Third-tier city□ Other□ | | |
