# Supplementary material for: Capability–environment configurations driving exploratory innovation in art entrepreneurs: A mixed-methods configurational study
Source: PLoS One. 2026 May 4;21(5):e0348315. doi: 10.1371/journal.pone.0348315 (PMC13138646; doi:10.1371/journal.pone.0348315)
Supplement: S4 File — (DOCX) [file pone.0348315.s004.docx]

# ------------------------------------------------

# Step 1: Install and load the NCA package

# (Run install.packages only the first time)

# ------------------------------------------------

install.packages("NCA")

library(NCA)

# ------------------------------------------------

# Step 2: Import dataset

# Replace the file path with your own CSV location

# Example path used here is only a placeholder

# ------------------------------------------------

data <- read.csv("D:/ResearchData/NCA_dataset.csv", header = TRUE)

# ------------------------------------------------

# Step 3: Effect size and significance test

# using CR-FDH ceiling technique

# X = column 1

# Y = column 7

# ------------------------------------------------

model_cr <- nca_analysis(

data,

x = 1,

y = 7,

ceilings = "cr_fdh",

test.rep = 10000

)

nca_output(model_cr, test = TRUE)

# ------------------------------------------------

# Step 4: Effect size and significance test

# using CE-FDH ceiling technique

# ------------------------------------------------

model_ce <- nca_analysis(

data,

x = 1,

y = 7,

ceilings = "ce_fdh",

test.rep = 10000

)

nca_output(model_ce, test = TRUE)

# ------------------------------------------------

# Step 5: Bottleneck analysis

# Analyze multiple necessary conditions

# X = columns 1–6

# Y = column 7

# ------------------------------------------------

model_bn <- nca_analysis(

data,

x = c(1:6),

y = 7

)

nca_output(

model_bn,

summaries = FALSE,

bottlenecks = TRUE

)
